# Conference Report: WORKSHOP ON CRITICAL ISSUES IN AIR ULTRAVIOLET METROLOGY Gaithersburg, MD May 26–27, 1994

**DOI:** 10.6028/jres.099.069

**Published:** 1994

**Authors:** Ambler Thompson, Mitchell K. Hobish

**Affiliations:** Radiometric Physics Division, National Institute of Standards and Technology, Gaithersburg, MD 20899-0001; 5606 Rockspring Road, Baltimore, MD 21209

## 1. Introduction

Several national programs and a variety of industrial applications today require the use or monitoring of ultraviolet (UV) radiation. The need for high accuracy radiometry in this region of the electromagnetic spectrum is becoming increasingly urgent. In response to this need, NIST’s Radiometric Physics Division organized a Workshop on Critical Issues in Air Ultraviolet Metrology. This workshop was held at NIST/Gaithersburg on May 26 and 27, 1994, immediately following the 1994 Council for Optical Radiation Measurements (CORM) meeting at NIST. The workshop was attended by more than 120 registered participants from industry, federal agencies, and academe. The goals of this workshop were to identify the needs of the UV measurement communities for standards and instrumentation necessary for absolute measurements of irradiance and radiance, compile a list of specific recommendations to improve the nation’s UV measurement capabilities, and determine NIST’s role in supporting these efforts. The Workshop was cosponsored by the Radiometric Physics Division of the National Institute of Standards and Technology, the Office of Research and Development of the Environmental Protection Agency, the Office of Polar Programs of the National Science Foundation, the National Renewable Energy Laboratory UV Monitoring and Assessment Program Panel, and the Cooperative State Research Service of the United States Department of Agriculture.

The complete ultraviolet spectral wavelength region is from 10 nm to 400 nm. Ultraviolet radiation is identified in working wavelength regions on both physical biological bases. The UV wavelength region designations on a physical basis are: the near UV (300 nm to 400 nm), the middle UV (200 nm to 300 nm), the far UV (100 nm to 200 nm) and the extreme UV (10 nm to 100 nm). The air UV region, or the UV region which air transmits is generally considered to be from 200 nm to 400 nm. Below 200 nm air is strongly absorbing and is termed the vacuum UV. The biological UV wavelength regions as defined by the Commission International De L’Eclairage (CIE) are: the UV-A (315 nm to 400 nm), the UV-B (280 nm to 315 nm), and the UV-C (100 nm to 280 nm). There is some confusion in the literature about the wavelength demarcation between the UV-A and UV-B regions, since the CIE has defined the demarcation as 315 nm and the more traditional breakpoint was 320 nm.

The workshop was organized to be tutorial in nature, showing the fundamental derivation of the UV measurement chain from basic physical radiometric standards, and the transfer of the radiometric measurement chain to specific applications, with attention to error propagation and uncertainty analysis. Due to the short duration and tutorial nature of the workshop, every important UV application could not be addressed. Because of the programmatic interests of the cosponsoring agencies, the second day of the workshop specifically showcased the radiometric needs for long-term solar UV monitoring and for medical and biological UV researchers. These application areas are of topical interest due to concerns over the increasing depletion of the stratospheric ozone layer.

The invited speakers were instructed to indicate the economic, social, or environmental importance of UV measurement applications, uncertainty requirements, problems in utilizing existing standards, and anything else important to the UV metrology communities. The workshop was organized into four oral presentation sessions, with four invited papers per session. The titles for the sessions were:

- Ultraviolet Radiometric Standards
- Radiometric Instrumentation, Calibration, and Measurement Uncertainty
- Measurement Requirements of Solar Ultraviolet Monitoring and Ozone Depletion
- Quantitation of Ultraviolet Biological Effects and Hazard Evaluations

An open forum discussion took place at the end of each session, during which details of that session’s presentations were discussed, and related issues raised.

Each of the sessions will be described below. The concerns raised at each individual session forum have been collated into a section on crosscutting issues, presented as a separate section, followed by a statement of workshop conclusions and directions for future work.

## 2. Opening Remarks

The workshop was opened by Dr. Katharine Gebbie, the Director of the NIST Physics Laboratory, who welcomed the attendees and provided background for the workshop by first describing the role of the National Bureau of Standards (NBS) from its inception in 1901 through to the Omnibus Trade and Competitiveness Act of 1988, as a result of which NBS became the National Institute of Standards and Technology (NIST) with broad new responsibilities. To further focus the Workshop, Dr. Gebbie described NIST’s Mission Statement generally, and the thrust of the Physics Laboratory specifically, with particular emphasis on UV metrology. Dr. Gebbie then discussed the Advanced Technology Program (ATP) in terms of its mission, strategy, and competitions, which are designed to increase the interactions between industry and government in (for the present) five focused areas: tools for DNA diagnostics, information infrastructure for health care, manufacturing composite structures, component-based software, and computer-integrated manufacturing for electronics.

A detailed introduction to the Radiometric Physics Division was then provided by Dr. Albert C. Parr, Chief of the NIST Radiometric Physics Division. Dr. Parr described the Division’s three primary goals: development, improvement, and maintenance of national standards and measurement techniques for radiation thermometry, spectroradiometry, photometry, and spectrophotometry; the dissemination of these standards; and the conduct of fundamental and applied research to support future measurement services. Dr. Parr provided an organizational overview of the Division, and then presented some details of the Division’s capabilities by describing the High Accuracy Cryogenic Radiometer, the Radiometric Calibration Chain, the Detector Comparator Facility, and the filter radiometer program. Dr. Parr closed with a discussion of the Division’s Calibration and Reference Material Programs, describing detector calibration, source calibration, and reflectance and transmittance capabilities. He noted that the Division is building the capability to perform material characterization in the infrared out to 25 μm in wavelength.

## 3. Session I: Ultraviolet Radiometric Standards

The first session was chaired by Mr. William E. Schneider from Optronic Laboratories, Inc. Dr. Donald Heath, from Research Support Instruments, Inc. presented the first of the tutorial talks. *Introduction to the Ultraviolet Spectral Region.* The approach was at first historical, referring to the work of Melvill (1752), Herschel (1800), Ritter (1801), Young (1802), Wollaston (1802), Fraunhofer (1814, 1823), Becquerel (1840), Kirchoff (1859), Angstrom (1868), Rowland (1887), Balmer (1885), Kayser, Runge, Rydberg (all 1890), Abbott (1923–1952), and Kiepenheuer (1930s). Dr. Heath also covered the phenomenological definitions of the UV regions: the air ultraviolet (200 nm to 400 nm), UV-A (315 nm or 320 nm to 400 nm), UV-B (280 nm to 315 nm or 320 nm), UV-C (200 nm to 280 nm). There is still not a consensus about the wavelength demarcation between the UV-A and UV-B regions Dr. Heath noted that the first measurements on extraterrestrial solar UV spectra were carried out by the Naval Research Laboratory in 1946 using spectrographs flown on captured V2 rockets. They observed that at 210 nm the solar irradiance was lower than expected from the Sun’s visible and UV blackbody temperatures; further into the UV, the solar emission exceeded that expected from the blackbody curve. Dr. Heath went on to describe solar ultraviolet radiation, and begin a detailed discussion of the solar spectrum. He opened up the topic of the effects of ozone (and its depletion) on UV levels measured at the Earth’s surface, thereby demonstrating the need for accurate UV data. To support the acquisition of these data, Dr. Heath opened a discussion on techniques for the calibration of UV spectroradiometric instruments, and provided some specific recommendations for radiometric calibration of UV surface radiation monitoring instruments.

The *Importance of Ultraviolet Measurements and Proposed Improvements to NIST*’*s Synchrotron Ultraviolet Radiation Facility (SURF II)* was the focus of the presentation made by NIST’s Dr. Robert Madden. Following the openings provided by Dr. Heath, Dr. Madden discussed the importance of high-accuracy radiometry by describing the needs of both national programs and industry in such measurements, with specific emphasis on the role of high-accuracy measurements in assessing the biological effects of UV-B radiation to human health directly, via skin carcinomas and melanomas, and the effects on food supplies, including both crops and ocean algae. Dr. Madden showed an action spectrum of the carcinogenic effectiveness of ultraviolet light, and data on the Antarctic ozone hole, emphasizing the need for measurements from instruments both above and below the ozone layer to monitor the incident and transmitted solar radiation, respectively. In support of the acquisition of high-accuracy data, NIST is making improvements to its SURF II, the characteristics of which are predictable from fundamental physics, and which will provide a continuum of radiation throughout the air and vacuum UV spectral regions. These improvements will fine-tune the electron orbital geometry in the storage ring, increase the energy of the stored electrons, and directly determine the radiant flux from the storage ring with a cryogenic radiometer. These improvements should achieve a radiometric uncertainty of less than 0.5 % in the absolute value of flux, and 0.1 *%* in its relative spectral distribution. The improvements should also extend NIST’s capabilities by providing spectral coverage to 2.4 nm, and should permit the determination of black body temperatures independent of gas thermometry.

The necessity for good, application-level UV measurements was addressed by Dr. Christopher L. Cromer, from NIST, in his presentation on *Ultraviolet Detector Metrology and Filter Radiometry.* Dr. Cromer first addressed the operational aspects of UV measurements with a description of the construction and workings of a typical UV meter. He stated that all too frequently, users employ such meters unmindful that the spectral range is generally not well defined. He demonstrated that large errors can be incurred either during calibration or through improper use. These errors could be due to the meter’s out-of-band rejection, nonlinearity, or non-cosine response. The calibration of the instrument and its use is dependent upon the spectrum of the calibration source and its similarity to the spectrum of the optical radiation to be measured. In addition, the relation of the sensor’s real response function to the ideal response function or desired action spectrum must be addressed. Dr. Cromer went on to describe source-based and detector-based calibration, NIST’s capabilities for UV detector metrology, and then suggested strategies for proper and effective calibration of UV meters.

The topic of proper calibration and suitability of standards for a given application was addressed by Mr. Robert D. Saunders, also of NIST, who presented material on *Choosing the Proper Standards for the Radiometric Application.* Mr. Saunders began by describing the parameters that need consideration, and, for the first time in the Workshop, addressed the need for portability and ease-of-use for standards and instruments. After describing the roles of primary, secondary, and working standards, Mr. Saunders went on to discuss the advantages and disadvantages of primary physical standards such as cryogenic radiometers, blackbodies, trap detectors, synchrotron radiation, parametric down conversion, and hydrogen arcs. A similar discussion of secondary and working standards followed, including 1000 watt quartz halogen tungsten lamps, D_2_ lamps, argon mini-arcs, tungsten strip lamps, and filtered detectors. Mr. Saunders then provided a look at the status of UV measurements, including the spectral irradiance intercomparison, and an ongoing air UV intercomparison, and showed how international irradiance scales compare with the NIST scale based on the international irradiance intercomparison performed in 1990.

## 4. Session II: Radiometric Instrumentation, Calibration, and Measurement Uncertainty

After a break for lunch, Session II was convened, with Dr. Theodore W. Cannon, from the National Renewable Energy Laboratory, as Chair. Dr. Cannon introduced Dr. Henry Kostkowski, of Spectroradiometry Consulting, who gave an excellent presentation on *Measurement Errors and Their Control in UV Spectroradiometry.* Dr. Kostkowski addressed the simple measurement equation, and showed how and under what conditions it could be employed. Discussion of the ideal responsivity function and actual responsivity (*R*) functions followed, with some guidelines on elimination of errors due to changing responsivity with direction and position through use of an averaging sphere, or the use of roughened quartz surfaces, which provide less attenuation. Things get more complicated when *R* changes with direction, requiring the use of a correction term in the measurement equation. Other errors are incurred (and must be corrected for) when *R* varies with polarization, the magnitude of the flux (nonlinearity), spectral scattering, distortion, drift, hysteresis, and wavelength instability. Dr. Kostkowski provided the group with some guidelines for choosing a UV spectroradiometer for high signal-to-noise ratios, and showed how the state-of-the-art could reduce uncertainties for solar terrestrial measurement at 295 nm by almost a factor of three. Dr. Kostkowski closed his talk with a nine-point plan for reliable spectroradiometry.

The need for calibration standards between laboratories was addressed in a presentation on the *NCSL: UV Radiometer Round-robin*, by Dr. L. Kasturi Rangan, of Lockheed Missile and Space Co., Inc. Dr. Rangan described a round-robin measurement program for UV irradiance among metrology standards laboratories under the auspices of the National Council of Standards Laboratories (NCSL). Fifteen laboratories-including NIST, the three primary standards laboratories of the DoD, and several aerospace companies–are participating. The circumstances which prompted this round-robin were discussed, as were the scope and status of the activity. Dr. Rangan presented information on the end-use of the UV sources measured by the calibrated meters, along with the manufacturers and models of those instruments. Further details for the instrumentation were provided, including the number of units calibrated by each laboratory per year, wavelength and bandwidth requirements, and irradiance levels. Five sources are to be used in the round robin along with several other optical components. Details on the UV round-robin parameters were presented, and a chart showing the uncertainty for each laboratory was shown. The circulating unit is now being calibrated at NIST and the estimated time for completion of the round-robin is on the order of 1 year.

Further information on *Spectral Ultraviolet Measurements and Utilization of Standards* was presented by Mr. William E. Schneider, of Optronic Laboratories, Inc. Mr. Schneider addressed instrumentation, standards, calibration, and measurement of optical radiation, and described the essential components of an automated spectroradiometric measurement system. Across his presentation were issues addressing the effects of wavelength accuracy and precision, linearity, slit function, stray light, scanning speed, cosine response, temperature dependence, and system calibration on the overall performance of the measurement system, with emphasis on the problems unique to measuring solar spectra in the ultraviolet. He described the construction and operational details of single and double monochromators of the Czerny-Turner variety, and showed a schematic diagram of a portable double monochromator spectroradiometer. After a discussion of general detector specifications, Mr. Schneider discussed signal detection systems, and proceeded to discuss various input optics, including none at all, cosine receptors, imaging optics, and fiber optics, with particular emphasis on an improved design for an integrating sphere. A short discussion of automatic data reduction systems followed, with a warning that operational details of these sometimes industry-standard components must be taken into account, as such a system is as much a part of the calibration and measurement chain as are standards and sources. Mr. Schneider then addressed calibration standards, particularly mercury arcs for wavelength standardization, and tungsten and deuterium lamps for spectral irradiance response standards. He then discussed the utility of plug-in irradiance standards, and ended with discussion of an automated spectroradiometer configured for measuring spectral irradiance.

The last of the formal presentations for Session II was by Mr. Daryl R. Myers, from the National Renewable Energy Laboratory, who discussed *The Uncertainty Challenge in Solar Terrestrial Ultraviolet Radiometry.* Mr. Myers reemphasized the importance of accurate and reproducible solar terrestrial ultraviolet metrology data, and so addressed his remarks to the problems associated with the calibration and measurement of solar terrestrial ultraviolet radiation between 280 nm and 400 nm. Using a standardized approach to uncertainty analysis, the sources and magnitudes of uncertainty in broadband and spectral UV radiometry were discussed, with examples of typical, currently available instrumentation, calibration sources, and techniques. Mr. Myers discussed the concept of acceptable uncertainty, and went on to describe in detail several sources of uncertainty, noting strongly the need to determine sources of uncertainty for all components in a measurement chain, making distinctions between random fluctuations which are reducible by increasing sample size, and systematic effects, some of which may be calibrated out by proper utilization of standards. Both types must be quantified and combined to yield total uncertainty for a measurement. Of great importance to the overall process is accurate reporting of uncertainties, so that workers in the discipline area may assess the validity of a measurement, given the real-world constraints of instrumentation.

A poster session was held at the Gaithersburg Hilton Thursday evening following the first day’s sessions. Some 15 posters were presented, representing several aspects of manufacturers’ progress and concerns in the production of measurement instrumentation, characterization and calibration of various instruments and networks, new mathematical corrections for radiometer data, and more. The poster session was well-attended, and resulted in significant, in-depth discussion among the participants.

## 5. Session III: Measurement Requirements of Solar Ultraviolet Monitoring and Ozone Depletion

The second day of the Workshop opened with Dr. C. Rocky Booth, of Biospheric Instruments, Inc., as Chair. The second day’s topics addressed specifics of applied measurements by researchers in the fields of solar UV monitoring, particularly as it applied to ozone monitoring and the biological effects of UV-B.

Dr. Booth introduced Mr. Ernest Hilsenrath, of NASA Goddard Space Flight Center, who discussed *Ultraviolet Calibration Requirements for Satellite Detection of Ozone, Solar Irradiance, and UV-B Trends.* Mr. Hilsenrath noted that the amount and spectral range of UV radiation reaching the Earth’s surface is a function of solar zenith angle, clouds, atmospheric turbidity, and the column amount of ozone. Changes in ozone, as a controlling factor for surface UV, becomes important only over the long term. To measure these long-term trends, NASA and NOAA have embarked on a program to monitor ozone and solar irradiance from space using the backscatter ultraviolet (BUV) technique. The program began in 1978 with Nimbus-7 SBUV/TOMS instruments. A national plan for ozone monitoring carries these measurements into the twenty-first century. These measurements will require accurate prelaunch calibration, careful monitoring, and precise on-orbit characterization. Corrections for albedo changes must be taken into account, but absolute accuracy relies on NIST’s standards to yield a calibration precision of 1 % at the lσ level. The uncertainty in an ozone measurement for 1 % calibration error varies, depending on the altitude. Mr. Hilsenrath concluded by noting that pre-launch activities require that absolute irradiance calibrations meet this uncertainty requirement and that absolute radiance calibrations based on BRDF and standard lamps are a problem. The problem of calibrations of multiple instruments is currently being studied and the requirement for post-launch instrument characterization to the 1 % level over long time periods is a major challenge.

Continuing the theme of global and regional UV level monitoring, Dr. C. Rocky Booth discussed *Calibration Aspects of the United States National Science Foundation*’*s UV Monitoring Network for Polar Regions.* This program, instituted in 1988, places a network of high spectral resolution (0.7 nm) UV spectroradiometers in locations around the globe, including Antarctica, Argentina, Alaska, and San Diego. These instruments are designed to be run in fully automated mode, and to provide continuous operation, 24 hours a day. The system is optimized for operation in the UV and visible spectral regions up to 600 nm. A vacuum-formed Teflon^®^ diffuser serves as an all-weather irradiance collector, and is heated to discourage ice and snow buildup. Provision is made for automatic wavelength and responsivity calibration 2–4 times daily. The instruments are comprised of two major subassemblies. The first includes the irradiance collector, monochromator, PMT and internal calibration sources. These components are mounted in a weather-proof enclosure, designed for mounting in the roof of existing structures. The second subassembly consists of power supplies, temperature controllers, electronic interfaces, and a personal computer. This group of components is usually mounted on a lab bench, away from the vagaries of weather. Dr. Booth described the details of calibration and characterization generally, and proceeded to discuss the data obtained by the network, which are available annually on CD-ROM, and will be made available over the Internet.

Dr. Brian Gardiner, of the British Antarctic Survey, commented on his being the only non-U.S. representative at this Workshop, and then proceeded to discuss *European Community Solar UV Spectroradiometer Intercomparisons: Review and Recommendations.* These intercomparisons were performed to improve the accuracy and reliability of solar ultraviolet spectral irradiance measurements in Europe. Over 3 annual campaigns, 14 different types of UV spectroradiometers have been studied. Each intercomparison campaign consisted of a variable number of instruments making simultaneous spectral measurements of the solar irradiance at one site over a range of observing conditions. These activities have resulted in improvements in instrument design, operational procedures, and increased the skill of the participants. The intercomparisons demonstrated that the best instruments show good agreement in their absolute irradiance calibration (although all instruments run into difficulties at the shortest UV wavelengths), but that there is still room for improvement in calibration techniques and procedures. These intercomparisons have also demonstrated the ability of workers to make plausible but still erroneous measurements. Based on laboratory measurements of slit functions and angular responses, the main areas of uncertainty in the irradiance measurements are scattered light in the calibration lamp room, accuracy of transfer lamp calibrations, temporal drift in the irradiance calibration of an instrument during the course of the day, and the effect of imperfect cosine and azimuth responses. Design and operational parameters for the better spectroradiometers include the use of double monochromator scanning spectrometers providing a spectral bandpass of 1.0 nm or less, and capable of making measurements every 0.5 nm in the range 280 nm to at least 420 nm.

Continuing the discussion of global-scale monitoring of UV levels. Dr. Betsy Weatherhead, of the Cooperative Institute for Research on Environmental Sciences/NOAA, opened her talk on *Ultraviolet Indexes and UV Monitoring Around the World* with a discussion of the need for such monitoring, and then provided a brief overview of UV instrumentation and an historical view of UV monitoring. This was followed by material on the purposes and outstanding problems of UV monitoring. She presented information on the locations of UV measurement stations around the world, and showed the near-exponential growth in numbers of spectral and broadband instrument stations from 1987 to the present. The difficulties in providing long-term, accurate, calibrated data sets are manifold, requiring calibration, documentation, careful analysis, and appropriate instrument placement and maintenance. These activities require large-scale coordination across organizational and national boundaries. Of particular importance is the need to communicate the results of such measurement campaigns to the public-at-large; this task is being addressed by the creation of UV indices. After presenting material on the definition of UV indices and their purposes, i.e., education of the public as to the effect of changing UV levels on their day-to-day activities and long-term health. Dr. Weatherhead went on to discuss the distinction between indices based on computer models and those based on measurements, discussing the advantages and disadvantages of both approaches. Real-world difficulties in generating such indices were discussed, including the differences between indices generated in different countries utilizing different criteria and parameters. Ultimately, representatives of the atmospheric, health, and educational communities must come together to define workable indices whose metrics will be usable regardless of geographical location.

## 6. Session IV: Quantitation of Ultraviolet Biological Effects and Hazard Evaluation

Session IV began when Dr. Martyn Caldwell, of Utah State University, was introduced by the session’s Chair, Dr. Edward DeFabo, of George Washington University Medical Center. Dr. Caldwell opened the discussion of biological effects of UV radiation by addressing *Ultraviolet Radiation Measurement Requirements in Terrestrial Plant Experiments*.

Understanding the mechanisms whereby biological systems are affected by environmental insults is not as clear-cut as understanding physical mechanisms of instrumentation response. To provide the best data in plant experiments both direct beam and diffuse ambient solar spectral measurements are required, as are spectral measurements of filtered solar radiation. Determining the action spectra of biological responses is necessary in order to determine what biomolecules are being affected by the incident radiation. Often, these spectra represent only a small portion of the incoming radiation spectral distribution. In most cases, action spectra for a given biological effect are different than for other effects. For example, the UV action spectra for the photoinhibition of the photosynthetic Hill reaction is significantly different from the action spectra for inhibition of ATPase, and action spectra for general DNA damage in stationary phase cells differs from induction of single strand breaks in DNA. As a result, care must be taken to fully define the spectral regions of incident UV radiation that are responsible for specific effects. To support this analysis, several different needs for UV measurements in plant experiments are necessary: spectral measurement of ambient solar radiation to establish benchmarks with solar radiation transfer models used to characterize baseline levels of UV-B radiation; spectral measurements of radiation used in experiments (from lamps, filtered solar radiation, etc.); continuous broadband measurements throughout the duration of experiments of ambient solar UV-B, UV-A, and visible radiation; and specialized measurements in some experiments, e.g., spectral irradiance within plant canopies. UV-B dosimeters have a significant role to play here, for continuous monitoring of solar and lamp radiation, routine checking of lamps and filter aging, and to check the spatial distribution of radiation fields. Other dosimeters may be used to monitor visible radiation, and for UV-A monitoring.

Continuing the discussion of biological responses to UV radiation. Dr. Robert M. Sayre, of Rapid Precision Testing Laboratories, discussed *UV Measurements in Photobiology and Photomedicine.* Ultraviolet sources are used for a variety of biologically related purposes, ranging from the treatment of disease conditions (such as psoriasis and atopic dermatitis), cosmetic uses (such as indoor tanning), drug phototherapy, efficacy testing of sunscreens and drug products, photostability testing of products and packaging, and as surgical and examination lamps. While some of these uses have had standards for measurement and usage developed, other photobiological uses are less well-defined, and are usually dependent upon specific action or response spectra. For sources covered by regulatory requirements, spectroradiometric measurements are required, and must be interpreted relative to a specific risk spectrum. Dr. Sayre discussed regulations, the nature of sources, and measurement requirements to support these uses. He made a strong case for long-term and ongoing monitoring of sources such as are used in tanning beds, as dosage is dependent on flux, which is known to vary with time. Further, Dr. Sayre amply demonstrated that many of the sources currently in use for photomedicine and photobiology and which are touted as providing reasonable representation of solar UV spectra do not, in fact, mimic those spectra with adequate fidelity. Specifically, Dr. Sayre concluded that specific standards for a given spectrum must be established, and that each source must be measured to insure that it meets that standard. Further, merely specifying a filter type is in-adequate, as manufacturers’ specifications often ignore out-of-band transmission. Sources must be described by spectroradiometric techniques; vague descriptions that a specific lamp was filtered with a specific filter are not adequate. Procedures must be developed for measuring sources, and these procedures must be standardized. Even in cases where laboratory measurement procedures have been standardized, it must be recognized that in-the-field use is not always congruent with laboratory procedures. Dr. Sayre asserted that educational programs must be established to train scientists in proper techniques for characterizing their sources, and to educate journal editors that sources are not generic.

Dr. Edward C. DeFabo, of the Laboratory of Photoimmunology and Photobiology at The George Washington University, carried some of the topics addressed by Dr. Sayre further in his presentation on *Skin Cancer, Immune Suppression, and UVB Radiation: Issues in Measurement.* UV-B has been specifically linked to skin cancer formation, and is also associated with local and systemic immunosuppression in mammals, although the mechanisms are not fully understood. The operational requirements of dealing with living, moving organisms provided significant challenges for dosage determination. To develop the action spectrum for immune suppression. Dr. DeFabo and his colleagues developed an optical dispersion system that could provide an area of exposure large enough to irradiate the dorsal surface of three mice at once, and to produce radiation with waveband resolution narrow enough (~2.5 nm) to allow for sufficient wavelength discrimination. This system was used to determine the absorption characteristics of the photoreceptor mediating immune suppression. In order to determine UV dosage, broadband and spectroradiometric measurements were utilized. Frequent calibration was required to accurately determine the absolute dose-response for immune suppression. The care taken in these studies allowed Dr. DeFabo and his colleagues to identify a unique photoreceptor on mammalian skin: a deamination product of histidine, an amino acid common to many proteins. This photoreceptor is commonly found in human skin, and may play a critical role in skin cancer development. The UV immune suppression phenomena have grave implications for susceptibility to contagious diseases and parasites due to the expected increase in solar ultraviolet irradiance projected to result from ozone depletion.

The question of correlating skin cancer occurrence with UV radiation dose was addressed by Dr. Martin A. Weinstock, of the Dermatoepidemiology Unit at Brown University. Dr. Weinstock discussed the *Epidemiological Correlations of Skin Cancer with Ultraviolet Exposure*, and described some of the adverse effects of sun exposure on human health. Specifically, Dr. Weinstock described malignant melanoma (MM), squamous cell carcinoma (SCC), and basal cell carcinoma (BCC). All three have been linked to sun exposure, although the nature of that linkage varies. An action spectrum has been determined for SCC, but not for the other types of cancer. Dr. Weinstock stated that precise geographic measurements of UV may assist in determining the other action spectra, and that these results will have significant implications for understanding the role of sunscreens, shade, and simple avoidance in the prevention of skin cancer. Dr. Weinstock emphasized that epidemiologists must distinguish between cultural shifts in behavior, e.g., clothing styles, and changes in environmental conditions, e.g., ozone depletion, in assessing skin cancer incidence trends.

## 7. Cross-Cutting Issues

Several themes kept arising throughout the presentations and during the discussions following each session. There was consensus that high-accuracy UV metrology was necessary to allow researchers to answer questions about biological effects of UV radiation and to monitor long-term environmental trends in such UV-related phenomena as ozone levels. To support this level of metrology, careful characterization of sources, filters, and instrumentation, as well as instrument intercomparison campaigns are required. It is not enough to say merely that a given source/instrument/filter combination was used, but rather calibration data must be provided in all reports. For a full understanding of the phenomena described above, the state-of-the-art in metrology must be extended well into the UV-C region. Common instrumentation needs included portability, ease-of-use, standardization of procedures, and improvement of integrating spheres.

The role of the various players in UV metrology was discussed at length, focusing most specifically on the national capabilities provided by NIST. With the increasing demand for high-accuracy radiometry, there is increased need for the secondary standards laboratories to provide for the transfer of NIST’s scales to the level of users’ needs. These secondary standards laboratories are private sector as well as governmental. A discussion of increasing use of secondary standards laboratories led to the conclusion that much, if not most, of the work of calibration and characterization needed for solar radiometry must be provided by NIST, EPA, NIH, NSF, DoE and other such groups, perhaps in the formation of core facilities for use by researchers in many communities and disciplines, thereby obviating the strong potential for duplication of effort.

Throughout the discussions ran the theme of education—of users, manufacturers, funding agency representatives, journal editors, and the general public. Users must be able to fully define their metrology requirements, matching required fluxes, accuracy, and precision with the instrumentation and sources needed to obtain suitable measurements. Users must also become better versed in the operational requirements of UV experimentation, and understand the relative uncertainties associated with each link in the measurement chain. Indeed, reports of experiments must include the calibrations described above, and a complete error and uncertainty analysis that includes all contributing factors, both qualitative and quantitative. Manufacturers must be educated about the needs of their end-user communities, including portability requirements, ease-of-use, and requirements for complete characterization of their instruments and sources to allow end-users to do top-quality experiments and measurements. Funding agency representatives must fully understand that when a researcher requests funds for an instrument of a given degree of precision and accuracy, that a less-expensive, less-accurate replacement will not suffice; research must be results-driven, not cost-driven. Journal editors must understand that full characterization of measurement chains is a *sine qua non* for publication of experimental and metrological results, for without such characterization it is almost impossible for other researchers to fully understand the measurements being presented, or to duplicate the work being reported. Finally, the public-at-large must understand that UV metrology can and will have increasing effects on their lives, as we struggle to understand how this energetic region of the electromagnetic spectrum affects biological systems.

## 8. Conclusions and Future Directions

In support of some of the issues raised during this Workshop, NIST has the ability to achieve accurate radiometric transfer from basic physical standards (primary standards) to detector or source secondary standards within their stated uncertainty. The use of these standards at their stated level of uncertainty requires that others understand the source under test, the standard, and the measurement instrumentation with a level of detail and proficiency approaching that of NIST’s scientists. Improvements in radiometric standards alone, uncoupled from a concomitant improvement in understanding of measurement instrumentation will not improve the accuracy of the nation’s measurement activities, as demonstrated by the results of the instrument intercomparisons and round-robins such as were reported on during this Workshop. The disagreement in measurements of a common source using a common standard are all-too-frequently inconsistent within the combined measurement uncertainty; therefore, improvements in the accuracy of the standards must be coupled to defining protocols and procedures for instrument intercomparisons and characterizations. There must be a strong educational component to the transfer of the radiometric measurement scales to applications. For important national programs and state-of-the-art-radiometry, NIST’s direct involvement may be necessary, but improvements can also be made through interactions with instrument manufacturers, secondary standards laboratories and measurement standardization organizations (i.e., Council for Optical Radiation Measurements (CORM), American Society for Testing and Materials (ASTM), and CIE).

The Workshop participants felt that this workshop was well organized and well run, and that it had already made a difference in the way many of them looked at their particular piece of the measurement puzzle. While industrial applications of UV radiometry were not represented in the body of the workshop presentations, there was strong representation by the industrial radiometry community in those who attended, which was an advantage provided by scheduling the workshop in proximity to the CORM94 meeting. A workshop to specifically target industrial applications of radiometry and photometry is being planned, as such input is required for the overall process of understanding the metrologic requirements of the radiometric community at large.

The material presented at this workshop will be collated and integrated into an Executive Summary, which will be published as a NIST Interagency Report by September 1994. The Workshop Proceedings will be published as a NIST Special Publication early in 1995.

